# Factorial and network structure of the Reynolds Adolescent Depression Scale (RADS-2) in Peruvian adolescents

**DOI:** 10.1371/journal.pone.0286081

**Published:** 2023-05-25

**Authors:** Cristian Ramos-Vera, Gleni Quispe Callo, Miguel Basauri Delgado, José Vallejos Saldarriaga, Jacksaint Saintila

**Affiliations:** 1 Research Area, Faculty of Health Sciences, Universidad César Vallejo, Lima, Peru; 2 School of Psychology, Universidad Nacional de San Agustín, Arequipa, Perú; 3 Postgraduate School, Universidad Femenina del Sagrado Corazón, Lima, Perú; 4 Escuela de Medicina Humana, Universidad Señor de Sipán, Chiclayo, Peru; University of the Basque Country UPV / EHU, SPAIN

## Abstract

Depression in young people is considered a public health problem, given that it affects their personal, social, and academic lives; therefore, early detection of depressive symptoms is of importance for a favorable prognosis. This study aimed to estimate the psychometric properties of the second edition of the Reynolds Adolescent Depression Scale (RADS-2) in Peruvian adolescents. The sample was composed of 917 Peruvian adolescents, aged 13 to 18 years (M = 15,241, SD = 1,020), who were selected from two public educational institutions in Metropolitan Lima. Confirmatory factor analysis supported the 25-item model with the four-dimensional structure and its overall and interdimensional reliability. This structure was found to be gender invariant. Finally, network analysis was performed to assess the relationships and centralities of the depressive symptoms of the validated version of the RADS-2. The results show that the RADS-2 measure is a consistent and reliable test that yields valid results in the Peruvian adolescent context.

## Introduction

Depression is considered as a multifactorial disorder and that groups behavioral, cognitive, social, biological symptoms [1], and emotional dysregulation [2, 3]. Depressive symptoms may begin in adolescence, a stage in which a variety of changes occur that place the adolescent in a situation of psychological vulnerability [4]. In fact, globally 34% of adolescents aged 10 to 19 years are at risk of clinical depression [5]. Moreover, according to World Health Organization estimates, approximately 1.1% of young people between 10 and 14 years of age and 2.8% of young people between 15 and 19 years of age suffer from depression [6]. Both depression and anxiety can have similar symptoms, such as sudden and difficult-to-predict mood swings [6]. Especially, in the school stage, depression is a cause or effect of academic aspects [7] that impact on school maladjustment, indiscipline in the classroom, problems with peers, and even reinforce a greater psychological vulnerability to other emotional, psychophysiological and behavioral disorders, such as comorbidity with dysthymia and generalized anxiety disorder [8, 9].

Although most depression screening instruments are applied to the adolescent population, they are based on a theoretical and psychometric model that responds mainly to the characteristics of adulthood, such as Patient Health Questionnaire-9 [10], Beck Depression Inventory II [11]. Except for the Children’s Depression Inventory-Short [12] which focuses on the 9 to 15 age group; similarly, the Reynolds Adolescent Depression Scale (RADS) [13], is a 30-item self-report questionnaire that assesses depressive symptomatology between the ages of 13 and 18 years. Unlike the aforementioned assessments, the RADS is based on the Diagnostic and Statistical Manual of Mental Disorders, Third Edition (DSM III) published by the American Psychological Association [14]. Originally, early validations conducted during the 1990s reported Cronbach’s alpha coefficient reliability values between .80 to .90 [15, 16].

In this sense, given the acceptance of the RADS at the time and the support of research that demonstrated the validity and reliability suitable for its application in the adolescent population, the RADS in its second edition (RADS-2) maintained the content of the items and to consider the significant characteristics of depression in adolescents, four theoretical dimensions were proposed: anhedonia/negative affect, dysphoria, somatic complaints, and negative self-evaluation, based on the DSM-IV TR and are consistent with the criteria for major depression of the DSM-V and the International Classification of Disease, eleventh edition (ICD-11) [17]. Additionally, the age range was extended from 11 to 20 years, with adequate internal consistency (α = .92) [13]. One of the differences of the RADS-2 with other measures that assess depression in the childhood context, such as the CDI, is the second version of the CDI (CDI 2) [18], or the summarized CDI (CDI-S) [19, 20], the latter revised in the Peruvian context, is that they consider dysphoria and negative self-esteem as central components. Whereas the RADS-2 can capture a greater variety of aspects of depression given that it maintains a greater number of factors that allow for a more complete and detailed understanding of the variable.

The four dimensions considered in the second edition are defined for a better understanding. The first dimension is made up of two constructs, anhedonia as that reduced capacity to feel pleasure [21] and on the other hand, negative affect involves different aversive states of distress, self-criticism and a tendency to have a negative view of oneself [22]. Dysphoria involves a state of emotional complexity characterized by discontent and unhappiness [23]. Somatic complaints are characterized by discomfort of a physical nature [24] that may be the result of a psychosocial phenomenon in the face of personal problems and disagreements [25]. Finally, negative self-evaluation involves regard for oneself, but marked by threatening thoughts and beliefs [26].

The validations of the current version of the RADS-2 assessed the internal structure by means of Confirmatory Factor Analysis (CFA). Osman et al. [13] conducted two studies with a sample of 458 adolescents, in the first study (N = 262) a bifactor analysis was conducted to assess the general and specific components of the RADS-2 and in the second (N = 196) a confirmatory factor analysis and divergent validity with other variables, both found acceptable reliability estimates (> .80). Likewise, in the study by Fonseca et al. [27], in a sample of 1,659 school adolescents, the tetrafactorial structure was validated.

Additionally, it has been adapted to other contexts, South Korea [28], Spain [29], United States [30], Iceland [31], New Zealand [32], and Sweden [17, 33]. In the case of Peru, the first adaptation was carried out on a sample of 1,963 students between 13 and 18 years of age from public and private educational institutions at the secondary level in Metropolitan Lima, which only used exploratory factor analysis to determine a six-factor structure [34].

Given that the psychometric properties of the RADS-2 have not been evaluated in Peru using the CFA, it is necessary to carry out this analysis with a greater methodological contribution to confirm the factors involved or to make the necessary modifications. This will provide a valid and reliable instrument for use in the detection of depressive symptomatology in adolescence, to prevent and in turn, intervene among adolescents with greater psychological vulnerability to emotional disorders [35]. Therefore, this research aims to evaluate the psychometric properties of the second edition of the RADS-2 in adolescents in the Peruvian context, such as internal structure, reliability and factorial invariance.

## Materials and methods

### Study design

The study is instrumental, with a cross-sectional approach, because it aims to analyze the psychometric properties of the RADS-2 through the CFA, reliability, and factorial invariance.

### Participants

For the calculation of the sample size, an a priori size calculator was used based on the structural equation model, with a minimum assumed effect size of .30, a probability level of .05, and statistical power of .95 [36, 37]. Therefore, the sample consisted of 917 students from two national elementary schools in the district of San Juan de Lurigancho in the city of Lima, Peru. Of the total, 464 were males and 453 females belonging to the secondary education level, whose ages ranged from 13 to 18 years (M = 15.241, SD = 1.020). The families residing in this district are in the middle, lower middle, and low socioeconomic levels [38].

### Instrument

Participants completed the Peruvian version [34] of the Reynolds Adolescent Depression Scale (RADS) [39], which assesses depressive symptoms in adolescents aged 11 to 20 years. It is characterized as a 30-item self-report, distributed in four dimensions (anhedonia/negative affect, dysphoria, somatic complaints, and negative self-evaluation). Participants completed a validated self-report measure of depressive symptomatology widely used in adolescent populations. The instrument assesses multiple dimensions of depressive symptoms and is administered using a Likert-type response format with ordered response categories. Following the standard scoring procedure of the instrument, a subset of indicators is reverse-coded. Higher total scores indicate greater levels of depressive symptomatology. Scores range from a minimum of 30 to a maximum of 120, where subjects who obtain higher scores on the instrument have greater depressive symptomatology, as opposed to those who maintain lower scores since they experience less depression.

### Procedure

For the application of the instrument, we used the previous adaptation of the RADS-2 for the Peruvian context [34]. The directors of two educational centers were contacted to gain access to the study participants with appropriate documentation mentioning the objectives of the research project and the significance of its execution. Consequently, parents or legal guardians and students were contacted to inform them of the reason for the investigation. Then, the voluntary participation of the participants aged between 13 and 18 years was requested prior written informed consent and assent. The study was conducted according to articles 24 and 57 of the Code of Ethics and Deontology of the College of Psychologists of Peru, 2017, which refer to the confidentiality of the personal data of the participants of the present study. Likewise, all procedures that contributed to the development of the study were carried out considering the ethical criteria of the 1975 Declaration of Helsinki and its subsequent modifications. The research protocol was approved by the ethics committee of Universidad Cesar Vallejo.

### Data analysis

The data analysis began with the descriptive results of the RADS-2 items, where the mean, standard deviation, skewness, and kurtosis values are reported, the last two of which must be within the interval of +/- 2 to consider univariate normality [40].

Once the descriptive results were identified, we proceeded to perform the CFA with the Diagonally Weighted Least Squares (DWLS) estimator due to the existence of ordinal data [41]. The original four-dimensional, 30-item RADS-2 model reported by Ugarriza et al. [34] was tested in Peruvian adolescents. Upon identifying that the factor loadings of five items did not exceed the value of .40, it was decided to eliminate them in order to obtain a better representativeness of the instrument [42], thus a model of the RADS-2 with 25 items and four dimensions was tested. In addition, to confirm both models, we reported the most common fit indices in instrumental studies, such as the fraction of chi-square and degrees of freedom (χ^2^/df < 4) [43], the comparative fit index (CFI > .90), the Turker-Lewis index (TLI > .90), the root mean square error of approximation (RMSEA ≤ .08), and the root mean square standardized residual root (SRMR ≤ .08) [44]. With the factor loadings, the omega coefficient was calculated for the RADS-2 globally and for each of the dimensions, such that when values above .70 were obtained, an acceptable reliability was considered [43].

Another analysis reported was the measurement invariance according to gender, in order to know if the instrument is equivalent for comparisons between men and women. First, the configural invariance was calculated where there are no restrictions and the factors are checked to ensure that they have the same pattern of free and fixed loadings. Then, the metric invariance is identified by constraining the factor loadings to be equivalent between the two groups. To check the scalar invariance, the intersections of the elements were restricted and for the residual invariance, we seek to restrict each of the residuals. Such analysis is performed progressively and is considered as acceptable by having adequate fit indices, likewise, to compare the different invariance models, the differences of ΔCFI < .01, ΔRMSEA < .03 and ΔSRMR < .03 were taken into consideration [45].

Psychometric network analysis was run with the R package qgrap with a LASSO estimator that allows spurious correlations to be suppressed [46]. Within the network, each of the RADS-2 items is included as nodes grouped into four factors, which are represented with a different color, and the lines that join the nodes are known as edges and determine the relationship that exists between indicators or symptoms [47]. Centrality was identified by means of the expected influence (EI) to see the importance of the individual nodes in the interconnection with the other variables represented in the system; as well as the expected influence bridging (IEB), which allows us to know the interconnection of the indicator with the other factors of the network [48]. This centrality was used because it is one of the most important and widely used in network analysis in psychology [49]. This network model considers the Fruchterman-Reingold "FR" algorithm [50] that refers to a greater stability and specificity of the statistical model [46].

## Results

Table 1 shows the descriptive results of the RADS-2, showing higher scores for two indicators, with a mean of 2.98 (SD = 0.89) and 2.51 (SD = 0.96) respectively. Skewness and kurtosis show univariate normality because their values are within the interval of +/- 2 [40].

**Table 1:** RADS-2 descriptive statistics.

Item	M	SD	g1	g2
Item 1	1.58	.75	1.26	1.30
Item 2	2.98	.89	−.50	−.57
Item 3	2.27	1.03	.14	−1.19
Item 4	1.68	.91	1.08	.03
Item 5	2.18	.98	.42	−.82
Item 6	1.92	1.00	.71	−.71
Item 7	2.19	.98	.22	−1.08
Item 8	2.11	1.01	.43	−.98
Item 9	1.90	.99	.72	−.71
Item 10	1.72	.89	1.11	.41
Item 11	1.88	.93	.79	−.34
Item 12	1.87	.92	.81	−.22
Item 13	1.76	.99	1.03	−.18
Item 14	1.46	.86	1.78	1.98
Item 15	1.82	.93	.82	−.40
Item 16	2.00	.92	.50	−.72
Item 17	2.21	1.02	.28	−1.08
Item 18	2.38	.98	.07	−1.02
Item 19	1.87	.96	.76	−.54
Item 20	1.68	.94	1.16	.19
Item 21	1.76	.96	.96	−.30
Item 22	2.59	.93	−.13	−.84
Item 23	2.05	.98	.61	−.63
Item 24	2.20	1.08	.34	−1.19
Item 25	1.70	.91	1.17	.42
Item 26	2.38	.95	.15	−.89
Item 27	2.10	1.00	.50	−.84
Item 28	2.51	.96	−.02	−.96
Item 29	1.72	.90	1.07	.20
Item 30	2.20	1.03	.34	−1.05

Note. M = mean; SD = standard deviation; g1 = Skewness; g2 = kurtosis

### Validity based on internal structure

Two models for RADS-2 were tested through the CFA, as shown in Table 2. The first model (M1) consisted of 30 items and four correlated factors, with factor loadings above .40 for most indicators. However, five indicators showed loadings below the recommended threshold, and were therefore removed to improve the representativeness of the instrument [42]. In the second model (M2), 25 items distributed in four dimensions were tested, with saturations above .40, which ranged from .55 to .82.

**Table 2:** Factor loadings of the proposed RADS-2 models.

Item	Model 1				Model 2			
	F1	F2	F3	F4	F1	F2	F3	F4
D2	.21				-			
D3	.63				.68			
D6	.62				.68			
D7	.71				.76			
D8	.69				.75			
D16	.60				.66			
D21	.72				.78			
D26	.55				.61			
D1		.78				.80		
D5		.62				.64		
D10		.31				-		
D12		.76				.76		
D23		.23				-		
D25		.26				-		
D29		.20				-		
D4			.56				.62	
D9			.74				.79	
D13			.68				.74	
D14			.66				.73	
D15			.61				.68	
D19			.62				.68	
D20			.76				.82	
D30			.66				.71	
D11				.48				.55
D17				.69				.74
D18				.56				.62
D22				.50				.57
D24				.49				.56
D27				.53				.60
D28				.51				.57
F1—F2	.55				.72			
F1—F3	.93				.96			
F1—F4	.93				.95			
F2—F3	.61				.75			
F2—F4	.40				.58			
F3—F4	.83				.83			

Note. Model 1 = 30-item model; Model 2 = 25-item model; F1–F4 represent the four latent factors identified in the confirmatory factor analysis.

Table 3 shows the fit indices for the models proposed for RADS-2, where a better fit is evident for M2 with values within acceptable parameters (χ^2^/df = 1.49, CFI = .996, TLI = .995, RMSEA [95% CI] = .023 [.018, .028], SRMR = .038). In addition, for the latter model, internal consistency indices were calculated through the omega coefficient for the general factor (ω = .93) and the four factors: dysphoria (ω = .83), anhedonia (ω = .70), negative self-evaluation (ω = .86), and somatic complaints (ω = .74).

**Table 3:** RADS-2 model fit indices.

Model	χ^2^/df	CFI	TLI	RMSEA [IC 95%]	SRMR
M1	2.45	.979	.978	.040 [.037, .043]	.052
M2	1.49	.996	.995	.023 [.018, .028]	.038

Note. χ^2^ / df = Chi-square/degree of freedom; CFI = Comparative fit index; TLI = Tucker-Lewis index; RMSEA = root mean square error approximation; IC = Confidence intervals; SRMR = Standardized root mean square residual. M1 = RADS-30 model; M 2 = RADS-25 model

### Factorial invariance

To demonstrate a greater equivalence of the RADS-2 according to gender, the factorial invariance of M2 was reported (see Table 4), because it has better adjustment indexes in the CFA. The invariance allowed us to sequentially and progressively calculate a series of models with different restrictions (configural, metric, scalar, and residual), such that if the latter models (scalar and residual) present considerable differences in the fit indices (ΔCFI < .01, ΔRMSEA < .03 and ΔSRMR < .03) [45], it is evident that the instrument does not have a measurement bias for the comparison of subgroups. Therefore, we first tested the unrestricted configural model (χ^2^/df = .96, CFI = 1.00, RMSEA = .00, SRMR = .044) to compare with the other restrictive models. Subsequently, metric invariance was found to have adequate fit indices (χ^2^/df = 4.01, CFI = .998, RMSEA = .014, SRMR = .048) with measurement differences within the stipulated parameters. Scalar invariance also had fit indices (χ^2^/df = 2.003, CFI = .999, RMSEA = .012, SRMR = .048) and differences (ΔCFI ≤ .001, ΔRMSEA ≤ .003 and ΔSRMR ≤. 000) considerable, as recognized for residual invariance (χ^2^/df = 1.46, CFI = .999, RMSEA = .009, SRMR = .048, ΔCFI ≤ .000, ΔRMSEA ≤ .003 and ΔSRMR ≤ .000). Based on the above, it is recognized that the M2 of the RADS-2 is invariant according to gender, therefore, the instrument is an acceptable measure to evaluate and make comparisons between men and women.

**Table 4:** RADS-2 measurement invariance by gender.

Model	χ^2^/df	CFI	RMSEA	SRMR	Δ CFI	Δ RMSEA	Δ SRMR
Configural	.96	1.00	.000	.044	-	-	-
Metrics	4.01	.998	.014	.048	.002	.014	.004
Scalar	2.00	.999	.012	.048	.001	.002	.000
Residual	1.46	.999	.009	.048	.000	.003	.000

Note. Δ CFI = Comparative adjustment index differences; Δ RMSEA = Mean square error difference of approximation; Δ SRMR = Standardized root mean square residual difference.

### Psychometric network analysis

**Fig 1:**
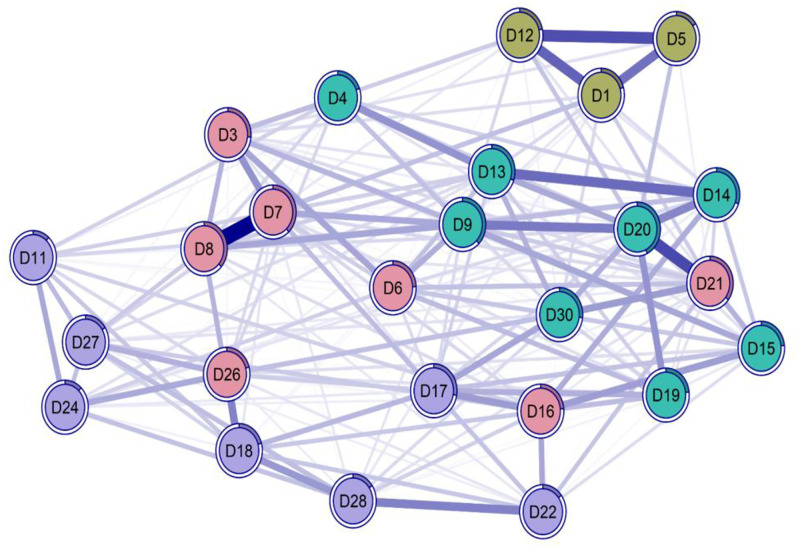
Network representation of item-level associations. Note. The figure illustrates a network visualization of item-level associations estimated using a psychometric network approach. Nodes represent individual observed variables, and edges represent partial correlations between them. Edge thickness reflects the strength of the associations. Node colors are used solely for visualization purposes and do not correspond to theoretical or diagnostic constructs. Blue paths are partial correlations between nodes. Red cluster = Dysphoria; Yellow Cluster = Anhedonia/Negative Affect; Green Cluster = Negative Self-Assessment; Cluster purple = Somatic Complaint. The rings of the nodes are the predictibilities of each node.

The network model (Fig 1) illustrated the pattern of associations among the RADS-2 indicators. Several indicators showed relatively stronger associations within the network, reflected in higher edge weights and predictability values. In contrast, other indicators exhibited lower predictability within the network structure.

**Fig 2:**
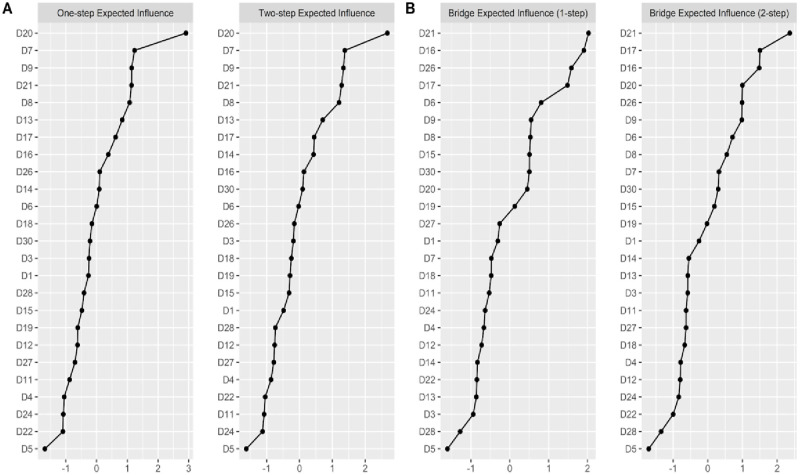
Network centrality indices of depressive symptom indicators. Note. z-scores are shown on the x-axis. A = Expected influence centrality of one-step (without considering clustering) and two-step (considering clustering); B = Expected influence centrality of one-step bridge (includes direct relationships) and two-step bridge (includes direct and indirect relationships).

Centrality analysis (Fig 2) based on one-step and two-step expected influence identified specific indicators with higher central influence and bridge influence across latent dimensions, whereas other indicators showed comparatively lower values across both metrics. These findings highlight heterogeneity in the contribution of individual indicators to the overall network structure.

## Discussion

The number of reported cases of adolescent depression is increasing [51] and is exacerbated in low- and middle-income countries [52]. Therefore, it is necessary to intervene early in those adolescent students with a higher risk of depression [53] to promote public policies focused on the prevention and treatment of depression in adolescents. Therefore, the present study sought to evaluate the psychometric properties of the RADS-2, considered one of the most widely used measures in the assessment of adolescent depressive symptomatology [54]. The results of the CFA show that the second model of 25 items with four factors presents a better fit of the fit indices. It refers to excellent general and interdimensional reliability indexes. It was also demonstrated that this structure is invariant to gender. This demonstrates that the proposed model has acceptable psychometric characteristics for use in adolescents in the Peruvian context, with the capacity to evaluate depression in male and female students without presenting biases in the understanding of each item.

Five items were eliminated from the instrument because they presented factorial saturations below the optimal cut-off point (< .40), which were done based on compliance with psychometric methodological rigor to avoid redundant information and lack of substantial contribution to the internal structure of the instrument [42]. Several items were removed from the instrument due to insufficient factorial saturation within the anhedonia/negative affect component. These items corresponded to reverse-coded indicators. Although item inversion is used to reduce the acquiescence effect [55], it is limited if the examinee does not have the necessary linguistic skills for comprehension, and may bias the psychometric analysis of the internal structure [56], consequently, the level of schooling may influence the assimilation of the statements and the respective response.

The findings of the validated factor structure were similar to other studies, for example, Blomqvist et al. [33], who worked with a sample of four schools from suburban and rural areas in Northern Sweden, identified that the CFA supported the four-factor model, however, they found item 23 to have a low load and pointed out that reduced communicative intentionality is a normal aspect of Swedes. In South Korea [28], it was reported that item 29 presented low factorial saturation in the RADS-2 tetrafactorial model and suggested that culturally Korean adolescents tend to worry too much about their body image or fear gaining weight. Skúlason and Freysteinsdóttir [31], assessed 541 Icelandic adolescents aged 12–16 years, and reported a good fit of the four-factor correlated model of the RADS-2, however, one indicator showed the lowest factor saturation on the anhedonia/negative affect factor. In the North American context, a non-clinical sample of 262 adolescents aged 14 to 17 years was evaluated and the bifactor structure (general factor and uncorrelated specific factors) was analyzed, showing questionable factor loadings among the five deleted items (coefficients from .00 to .12) in the general factor with the highest variance explained (31.89%) compared to the specific factors [13].

Additionally, one of the indicators was assigned to the Dysphoria subscale, resulting in a low factor loading, which can be explained based on the theoretical divergence between school anxiety and dysphoria, understanding school anxiety as those cognitive, psychophysiological and motor responses that are emitted by the student when perceiving certain school situations as threatening [57], while dysphoria implies a disturbance in mood that includes a feeling of loneliness and irritability in daily activities, as referred by Reynolds [58]. This finding is similar to the psychometric study of the RADS-2 by Fonseca et al. [27], the four-factor correlated model was corroborated, where item 2 presented the lowest factorial coefficient (.17), as well as weak factorial saturations (< .40) with respect to the other items eliminated in the present study.

The dimension formed by Anhedonia and negative affect presented several indicators with reduced factor loadings, which were not considered in the short version of the scale, with the exception of one indicator reflecting reduced affect that presented an adequate factor loading (> .40) [59]. In addition, other indicators demonstrated adequate factorial saturation and were consistent with the Negative Affect component, demonstrating its representativeness in depressive symptomatology, considered as a common factor in mood disorders [60]. It can be explained that the deleted items corresponding to the anhedonia factor did not contribute satisfactorily to the measurement of the adolescent depression construct, as this component allows for the assessment of depression in adults, as confirmed by the DSM-5 by replacing the criterion of depressed mood or anhedonia with irritability as a core diagnostic symptom criterion in adolescents [61]. Certainly, in the context of the metropolitan area of the city of Lima, this negative evaluation of oneself reflects a perception of failure and insecurity, which is characteristic of adolescents with a higher prevalence of depression [62].

On the other hand, several previous investigations maintained the 30 items of the RADS-2, for example, a study conducted in Pakistan where 330 school adolescents aged 11 to 20 years from the city of Karachi were evaluated, endorsing the model of four correlated factors, approving its use in the Pakistani population [63]. In Spain, a factor analysis of the RADS-2 was performed in a sample of adolescents from Barcelona (N = 1.384) who reported ages between 11 and 16 years, where the CFA supported the parsimonious model of four correlated factors, whose optimal factor structure refers to standardized coefficients between .67 and .97 at the item level [29].

### Invariance by gender

The study evaluated the invariance of the RADS-2 in groups corresponding to gender, finding that the scale was invariant in relation to all levels (configural, metric, scalar, residual). Other studies evaluated factorial invariance according to gender and age. The first one was performed by Blomqvist et al. [33], identifying RADS invariance at the constraint levels (configural, metric and scalar). The second study by Ekbäck et al. [17] evaluated a clinical sample of patients belonging to child and adolescent psychiatry, also demonstrating invariance at 3 levels and additionally between clinical and non-clinical samples. On the other hand, Fonseca et al. [27] showed the invariance of the levels (configural, metric) between gender and age groups. Even the invariance of the instrument has been determined in other factor models [59, 64, 65].

Since not all studies have been able to identify the invariance of the four levels, the present study is important in demonstrating the overall invariance and comparing the differences in means according to gender. Thus, the importance of invariance lies in recognizing the feasibility of comparing concepts of interest between groups [66] and the participants interpreting the items in a given latent factor in the same way [67].

### Network analysis

Additionally, a systemic model of RADS-2 symptom interaction is evaluated by means of psychometric network analysis. Within the network structure of the model, it is shown that one indicator had the greatest central influence and variance explained, this indicator has also been identified as a core measure in other network findings of depressive symptomatology in 1.409 American adolescents [68], 5.888 adolescents from the Netherlands [69], and 10.233 Korean students [70]. Recently, during the context of COVID-19, similar results of network centrality of self-contempt have been reported in several studies conducted in Chinese adolescents [71, 72].

One indicator related to sadness was also one of the most central within the network, as is the case with another closely related indicator, which usually precedes sadness because they arise in contexts of great emotional intensity [73], these indicators were reported to be more influential in another network research of depressive symptomatology in adolescents aged 10 to 17 years [72]. An additional indicator related to negative affect of the negative affect factor is also considered as a core indicator within the network structure and is another indicator found to be related to sadness. Since people who perceive themselves as rejected or undervalued within different social groups tend to maintain negative experiences that involve a cognitive and emotional change that increases states of sadness and depressive feelings [74]. Another network study in Spanish adolescents reinforces the importance of the interconnectedness of these indicators in systemic depressive symptomatology [75]. It is likely that these symptoms (not feeling loved and sadness) are more prevalent in those with family communication problems, as evidenced in the research of Zhou et al. [72], where such symptoms were more related to lack of assertive family communication.

Ekbäck et al. [17] reported that item 21 presented lower representativeness in the internal structure of the RADS-2, while in the present study it was identified as one of the most influential symptoms (higher centrality-bridge index) in the interconnective comorbidity of depressive symptomatology in Peruvian adolescents. This may indicate that the feeling of compassionate and sincere pity towards oneself is more relevant in the Peruvian context, where adolescents in the face of greater emotional distress tend not to consider themselves worthy of love or compassion and even tend to victimize themselves [76]. According to the findings of the network, it is probably due to its location as a nexus and being closer to the symptoms linked to negative self-image such as self-contempt, low self-esteem, and hopelessness.

Another of the findings of greatest clinical interest given its greater comorbid interconnectedness is one indicator characterized by pessimistic tendencies, which has been reported as one of the most central indicators in the network results of patients with a depressive diagnosis [77–79]. Is more likely to interconnect with other symptoms belonging other depressive domains of negative affect and dysphoria, since it has a stronger relationship with the symptomatology of related affective and behavioral indicators. Adolescents who show a pessimistic view of the future come to experience greater hopelessness because they lack the ability to set goals and maintain a gloomy expectation that the events that may occur are negative, accompanied by the belief that they cannot do anything to change these events [80]. In addition, subjects with greater feelings of sadness have a lower ability to tolerate and modulate stressful events, which are often related to a covert hostility that manifests itself in feelings of guilt, self-loathing, resentment and irritability [81].

### Limitations

The limitations of the present study are related to representativeness, since in relation to accessibility only public institutions were considered in the study, which makes it difficult to extrapolate to the context of private education or other regions different from Lima. In addition, the research is cross-sectional and to increase the evidence regarding the predictive validity of the instrument, a longitudinal study would be more advisable, as well as including a test-retest in the reliability analysis. The strength of the network analysis is that it provides a complementary and significant approach to the understanding of depressive symptomatology from the estimation of the network of relationships of all components [82].

## Conclusion

In conclusion, the results show acceptable psychometric characteristics for use in the adolescent population in the Peruvian context, supporting both the factorial structure of the four dimensions and the reduced item set, as well as measurement invariance according to gender. It is recommended to further study the psychometric properties in the clinical adolescent population and other regions of Peru, as well as to continue with the analysis of the factorial invariance of the instrument according to age, ethnicity, socioeconomic level, among others.

## Supporting information

S1 Data Reynolds Adolescent Depression Scale. (CSV)
