# Correction: Factorial and Network Structure of the Reynolds Adolescent Depression Scale (RADS-2) in Peruvian Adolescents

**DOI:** 10.1371/journal.pone.0346101

**Published:** 2026-03-30

**Authors:** Cristian Ramos-Vera, Gleni Quispe Callo, Miguel Basauri Delgado, José Vallejos Saldarriaga, Jacksaint Saintila

## Notice of Republication

This article was republished on March 11, 2026 to remove copyrighted material. Please download this article again to view the correct version.
